# Supplementary material for: A Cyclic Pentamethinium Salt Induces Cancer Cell Cytotoxicity through Mitochondrial Disintegration and Metabolic Collapse
Source: Int J Mol Sci. 2019 Aug 28;20(17):4208. doi: 10.3390/ijms20174208 (PMC6747461; doi:10.3390/ijms20174208)
Supplement: Supplementary file 1 [file ijms-20-04208-s001.pdf]

Synthesis and Characterization of compound 1-3C

Synthesis:

The flask was charged with 2-methyl-3-propyl benzothiazolium iodide 180 mg (0.56 mmol), 2-(2-Quinoxalinyl)malondialdehyde 50 mg (0.25 mmol), and dry buthanol (7 mL). The mixture was stirred at 105°C for 18 h. After cooling to laboratory temperature, the mixture was filtered and the solid part was washed with buthanol (3x3 mL), with ethanol (3x5 mL) and finally washed with diethylether (2x10 mL). Obtained solid was dried in vacuo. The product **1-3C** was obtained as greenish powder, 136 mg, 81 %.

Characterization:

^{13}C NMR (126 MHz, DMSO-d_6, 25 °C): 169.70, 158.24, 148.96, 147.88, 144.09, 143.22, 141.19, 140.50, 139.10, 138.61, 129.05, 128.81, 128.45, 128.27, 128.17, 127.91, 127.82, 126.92, 126.50, 125.66, 123.81, 123.19, 119.05, 115.32, 114.12, 106.37, 103.76, 49.79, 49.42, 21.50, 20.38, 11.33, 10.83.

^{1}H NMR (500 MHz, DMSO-d_6, 25°C): 8.65 (1H, br s), 8.49 (1H, br d, 14.5 Hz), 8.33 (1H, d, 14.5 Hz), 8.19 (1H, d, 7.8 Hz), 8.18 (1H, d, 7.7 Hz), 8.14 (1H, d, 8.3 Hz), 8.13 (1H, d, 8.1 Hz), 8.06 (1H, d, 8.1 Hz), 8.01 (1H, d, 8.2 Hz), 7.78 (1H, m), 7.75 (1H, m), 7.73 (1H, t), 7.66 (1H, t, 7.7 Hz), 7.61 (1H, t, 7.6 Hz), 7.47 (1H, t, 7.5 Hz), 4.78 (2H, t, 7.4 Hz), 4.65 (2H, t, 7.2 Hz), 2.06 (2H, m), 2.03 (2H, m), 1.22 (3H, t, 7.4 Hz), 1.20 (3H, t, 7.4 Hz).

HRMS Calculated for $C_{33}H_{29}N_4S_2^+$ 545.1828, Found: 545.1824.

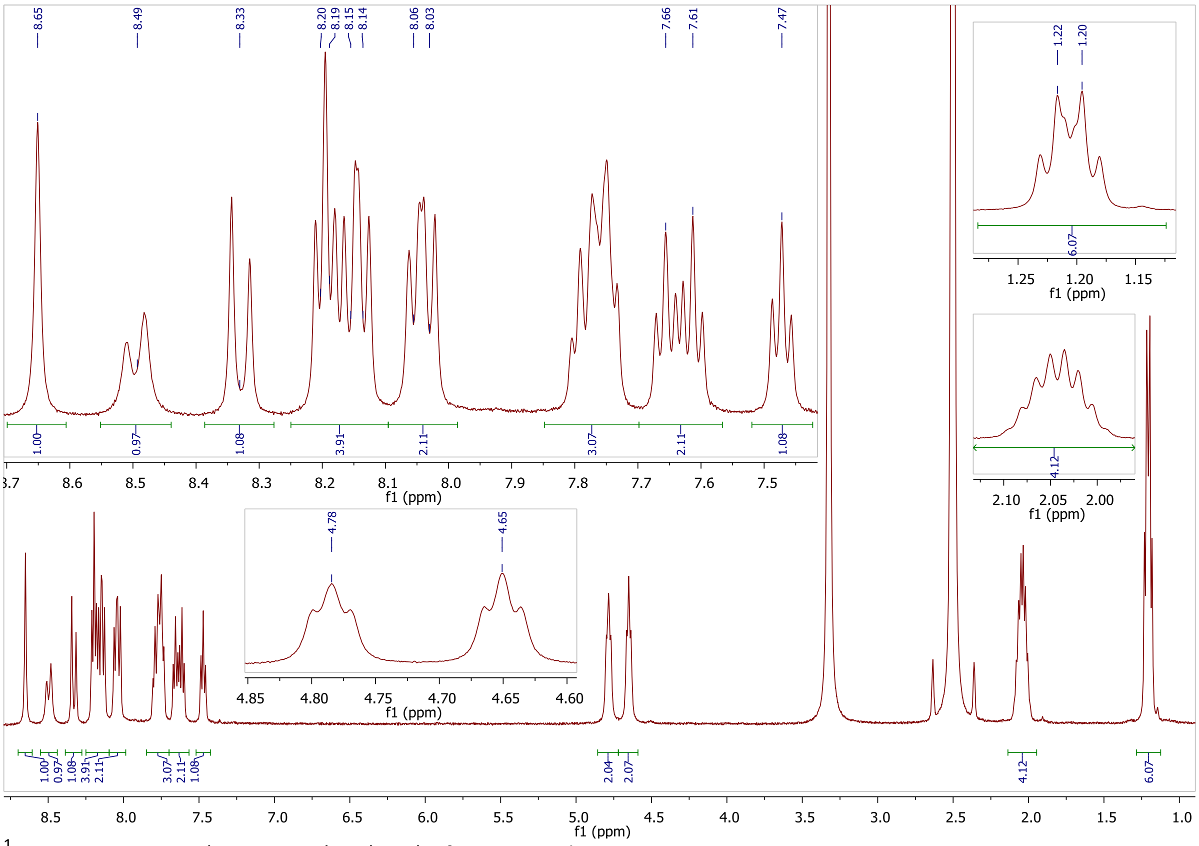

^{1}H NMR spectrum (500 MHz, $(CD_3)_2SO$) of compound **1-3C**

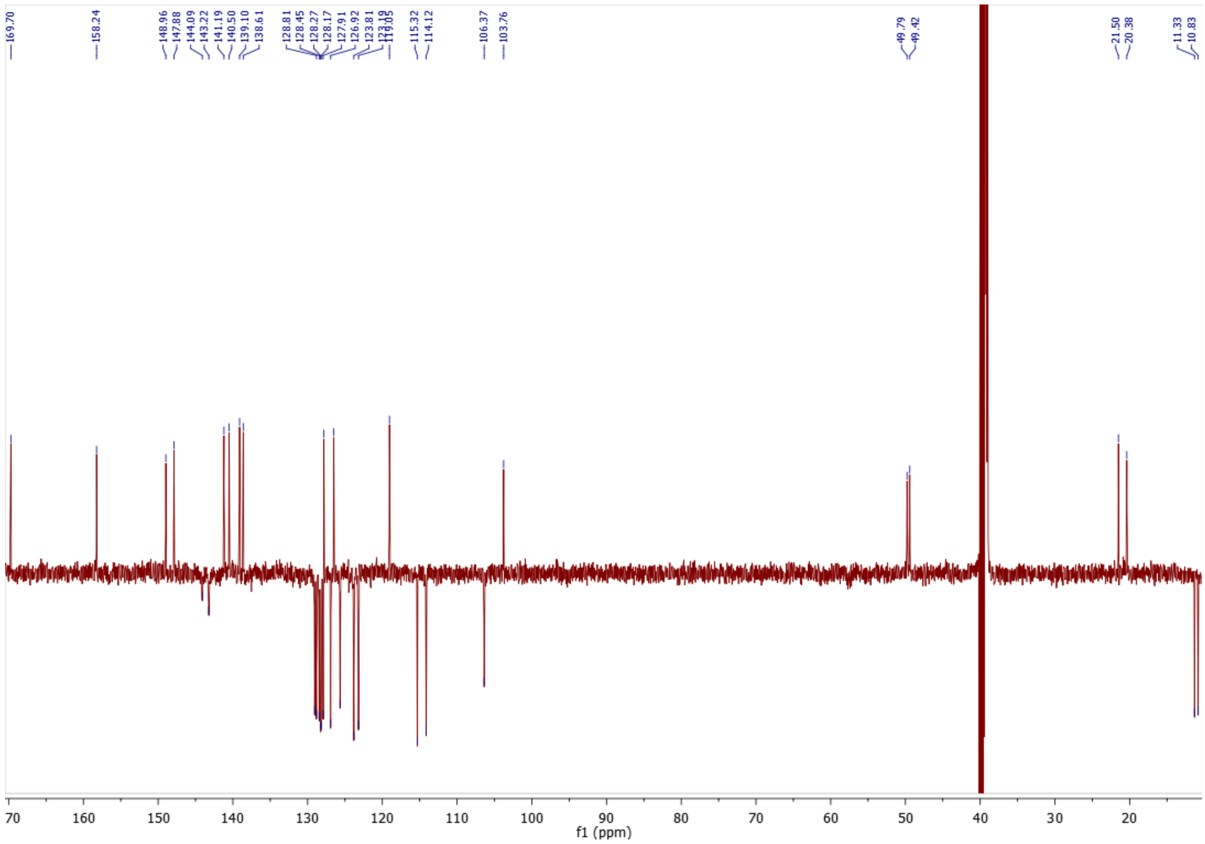

^{13}C APT NMR spectrum (126 MHz, $(CD_3)_2SO$) of compound **1-3C**

Synthesis and characterization of compound 1-8C

Synthesis:

Heteroaromatic salt *N*-octyl-2-methyl-benzothiazolium iodide 747 mg (1.92 mmol), 2-(2-Quinoxalinyl)malondialdehyde 180 mg (0.90 mmol) and dry buthanol (15 mL) were added to the 50 mL flask. The mixture was stirred and heated to 105°C over 18 hours. After this time, the product was filtered off, washed with buthanol (2x3 mL), with ethanol (1x5 mL) and finally washed with diethylether (2x10 mL). Obtained solid was dried in vacuo over night. The product was obtained as a metalic greenish solid, yield: 247 mg, 34%.

Characterization:

^{1}H-NMR (500 MHz, DMSO) δ 8.56 (s, 1H), 8.39 (d, 1H, *J* = 14.4 Hz), 8.21-8.09 (m, 5H), 7.97 (m, 2H), 7.74 (m, 2H), 7.68 (m, 1H), 7.61 (m, 2H), 7.44 (t, 1H, *J* = 7.5 Hz), 4.76 (t, 2H, *J* = 7.7 Hz), 4.60 (t, 2H, *J* = 7.7Hz), 1.93 (m, 4H), 1.57 (m, 4H), 1.39 (m, 4H), 1.25 (m, 8H), 1.14 (m, 4H), 0.83 (t, 3H, *J* = 6.4 Hz), 0.70 (t, 3H, *J* = 6.9 Hz).

^{13}C NMR (126 MHz, DMSO) δ 169.39, 158.12, 148.84, 147.75, 143.98, 142.99, 140.93, 140.31, 139.03, 138.55, 128.90, 128.80, 128.42, 128.13, 127.77, 127.52, 126.90, 126.43, 125.60, 123.76, 123.13, 118.99, 115.22, 113.99, 106.04, 103.76, 48.48, 47.96, 31.22, 31.05, 28.72, 28.64, 28.61, 27.90, 26.77, 26.37, 25.85, 22.09, 22.03, 13.95, 13.77.

HRMS Calculated for $C_{43}H_{49}N_4S_2^+$ 685.3393, Found: 685.3397.

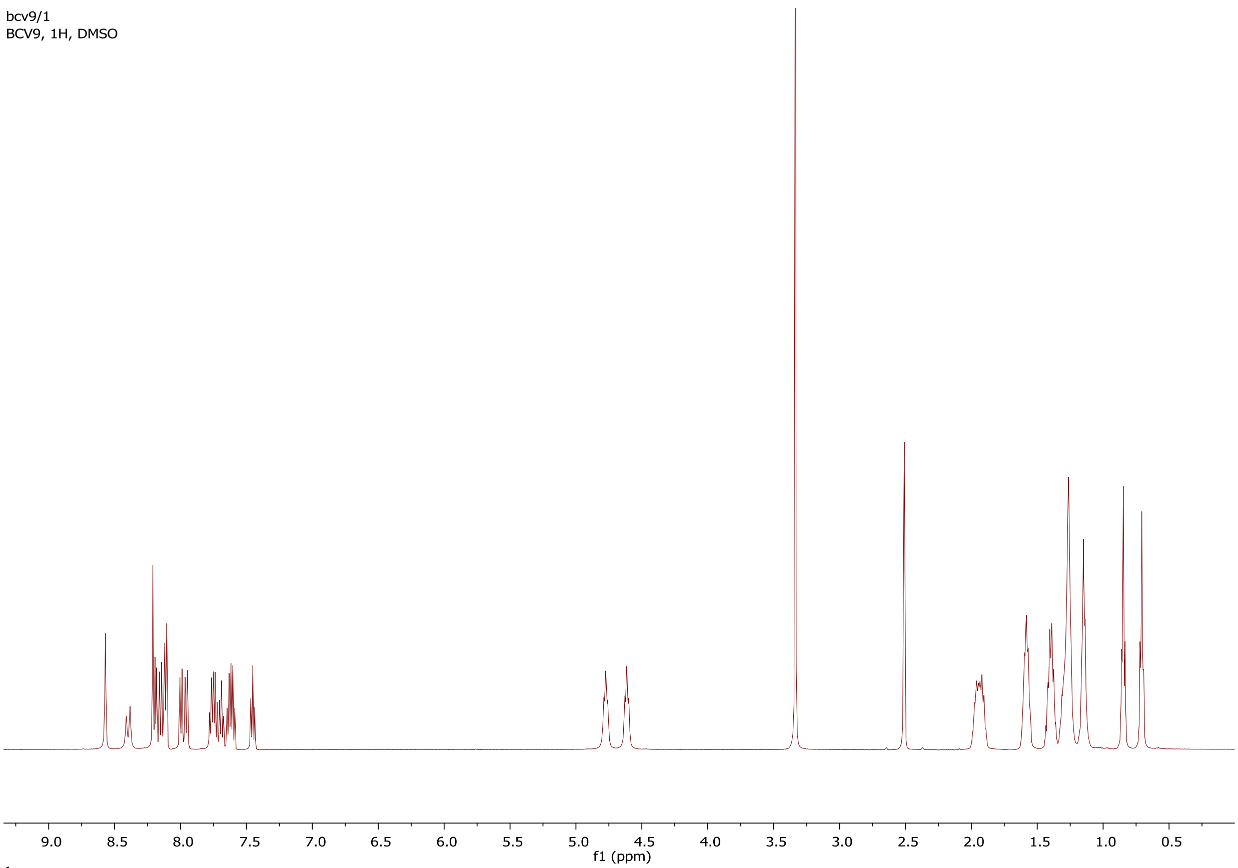

^{1}H NMR spectrum (500 MHz, $(CD_3)_2SO$) of compound **1-8C**

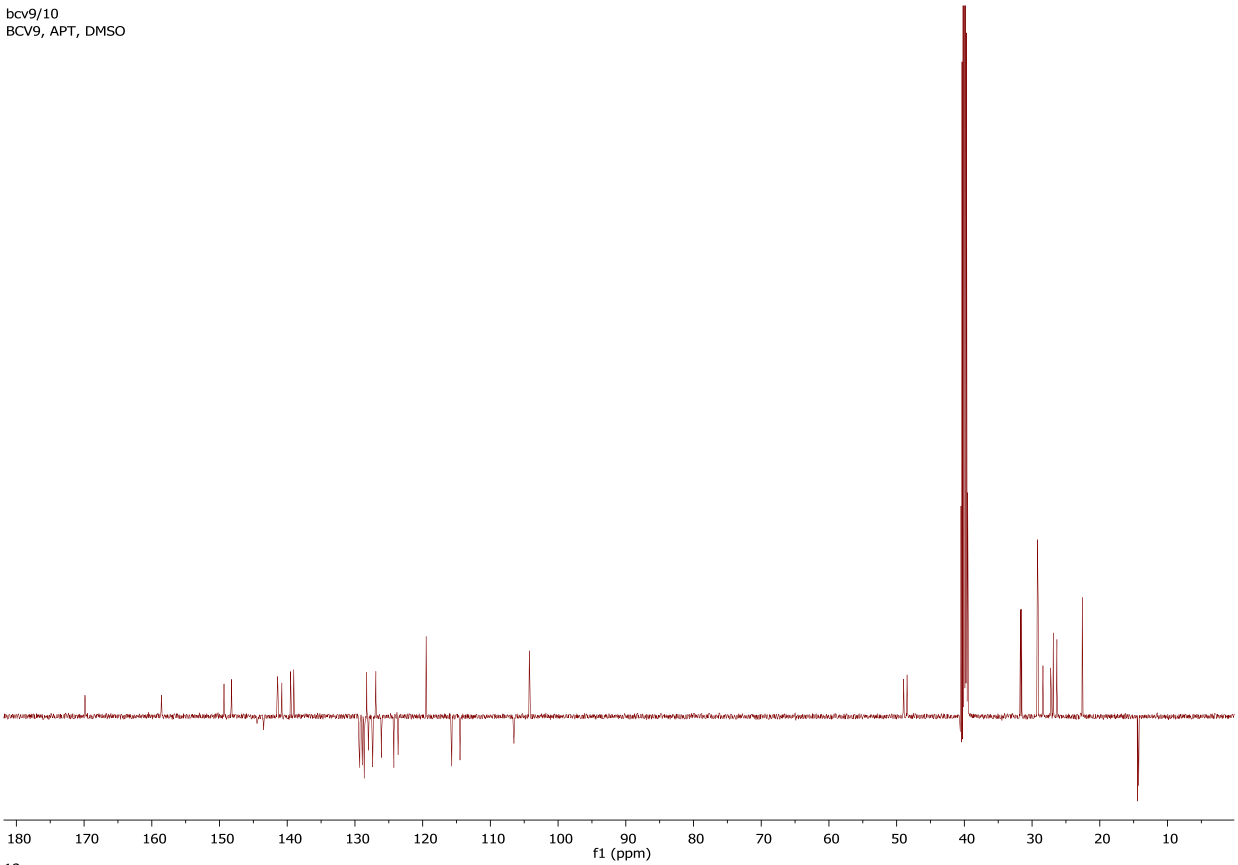

^{13}C APT NMR spectrum (126 MHz, $(CD_3)_2SO$) of compound **1-8C**
